# Preoperative short- vs. long-course chemoradiotherapy with delayed surgery for locally advanced rectal cancer

**DOI:** 10.18632/oncotarget.10280

**Published:** 2016-06-24

**Authors:** Mi Joo Chung, Dong Wook Kim, Weon Kuu Chung, Suk Hwan Lee, Seung- Kyu Jeong, Jae Kwan Hwang, Choon Sik Jeong

**Affiliations:** ^1^ Department of Radiation oncology, Kyung Hee University Hospital at Gangdong, College of Medicine, Kyung Hee University, Seoul, Korea; ^2^ Department of Surgery, Kyung Hee University School of Medicine, Seoul, Korea; ^3^ Department of Surgery, Yang Hospital, Seoul, Korea; ^4^ Department of Surgery, Yang Hospital, Namyangju, Korea; ^5^ Department of Surgery, Hansol Hospital, Seoul, Korea

**Keywords:** chemoradiotherapy, preoperative treatment, rectal cancer

## Abstract

**Purpose:**

To compare the clinical outcomes between short-course chemoradiotherapy (CRT) and long-course CRT with delayed surgery in locally advanced rectal cancer patients.

**Results:**

From 2010 to 2015, 19 patients were treated with short-course CRT and 53 patients were treated with LCRT. The sphincter-saving rate (89.5% vs. 94.3%, short-course CRT vs. long-course CRT), pathologic complete remission (21.1% vs. 13.2%), downstaging (47.4% vs. 26.4%), and treatment complications including anastomotic site leakage, bowel adhesion, and hematologic toxicity associated with short-course CRT were not significantly different from those associated with long-course CRT. 2-year overall survival was 90.0% and 91.2% (*p* = 0.448), respectively.

**Methods and materials:**

72 patients with stage cT3-4N0-2M0 rectal cancer participated in a multicenter study. Short-course CRT treatment was as follows: a total of 25 Gy of radiotherapy was delivered in 5 equal doses with intensity modulated radiation therapy. Chemotherapy was consisted of Leucovorin 400 mg/m^2^ administered by bolus injection on day 1 and 5-Fluouracil 1200 mg/m^2^ given by continuous infusion on days 1 and 2. An additional three cycles of chemotherapy were administered before the surgery. Long-course CRT treatment was as follows: a total of 50.4 Gy of radiotherapy was delivered in 28 equal doses. Chemotherapy consisted of a bolus injection of 5-Fluouracil + Leucovorin during the first and last week of radiotherapy. Surgery was performed 6−8 weeks after completion of radiotherapy in both groups.

**Conclusions:**

Preoperative short-course CRT is an effective and safe modality. It is clinically comparable to long-course CRT in locally advanced rectal cancer.

## INTRODUCTION

Preoperative conventionally fractionated chemoradiotherapy(CRT) has been adopted as a standard treatment for patients with stage II or III rectal cancer.

The German Rectal Cancer Study Group reported that preoperative CRT improved local control and reduced treatment-associated toxicity in comparison with postoperative CRT [1]. In addition, the Dutch Colorectal Cancer Group and the Swedish Rectal Cancer Trial demonstrated the value of short-course radiotherapy (5 × 5 Gy). Both trials reported that short-course radiotherapy led to a reduction in the risk of local recurrence [2].

However, short treatments may preclude the chance of sphincter-preserving surgery, since tumors shrink slowly [3–7]. In addition, complete remission as reported in a Polish trial and in the Tans-Tasman Radiation Oncology Group Trial (TROG) 01.04 was significantly higher after CRT than after short-course radiotherapy [8].

Thus, the optimal radiotherapy regimen is a source of contention. The timing of surgery after short-course radiotherapy is another concern. Latkauskas et al. presented a randomized controlled trial comparing patients with rectal cancer treated with short-course radiotherapy and long-course CRT, both with delayed surgery [9]. The R0 resection rate and sphincter-preserving surgery rate were not different between the two groups, but pathologic complete remission in the short-course radiotherapy group was lower than that in the long-course CRT group.

Adding concurrent chemotherapy to radiotherapy has been shown to improve pathologic response rates [10]. So, chemotherapy was administered concurrently with short-course radiotherapy in our institution.

The purpose of the present study was to evaluate the pathologic response rates and sphincter-sparing surgery rates achieved after short-course CRT compared with those after conventional long-course CRT.

## MATERIALS AND METHODS

### Patient selection

Eligible patients had a histopathologically confirmed adenocarcinoma within 15 cm of the anal verge, clinical stage T3-4N0-2M0 cancer, and no history of previous malignancy. We enrolled 72 patients with rectal cancer who underwent preoperative CRT and curative surgery between March 2010 and June 2015.

All patients underwent staging workups including digital rectal examinations, flexible endoscopies, abdominal and pelvic computed tomography scans, complete blood counts, liver enzyme assays, and serum CEA analysis. If further workup was needed, magnetic resonance imaging or positron emission tomography scanning of the pelvis and liver were performed. Clinical and pathologic stages were determined according to the American Joint Committee on Cancer Staging, 7th edition. The definition of a pathologic complete response (ypCR) was: lack of viable tumor cells, and a fibrotic mass only in the pathologic specimen. Downstaging was described as ypT0-2N0.

According to each particular surgeon's preference for RT regimens, patients were given long course CRT or short-course CRT. Most patients who lived a long distance from the hospital were given short-course CRT.

### Treatment

All patients received preoperative CRT. Radiotherapy was delivered with the patients in a prone position, using a belly board. Target volumes and organ at risk were contoured according to the guidelines of the International Commission on Radiation Units and Measurements Report 50.

Radiotherapy consisted of a total pelvic dose of 45 Gy and a booster dose for the primary tumor up to 50.4 Gy; chemotherapy was a bolus injection of 5-fluorouracil and leucovorin (FL) in the first and last week of radiotherapy for the long-course CRT group.

In short-course CRT, a total dose of 25 Gy radiotherapy was administered in 5 fractions of 5 Gy with an intensity modulated radiotherapy. Chemotherapy was delivered with radiotherapy and was consisted of leucovorin 400 mg/m^2^ administered by bolus injection on day 1 and 5-Fluouracil 1200 mg/m^2^ given by continuous infusion on days 1 and 2. Before surgery, 3 cycles of chemotherapy were done at fortnightly intervals.

**Figure 1:**
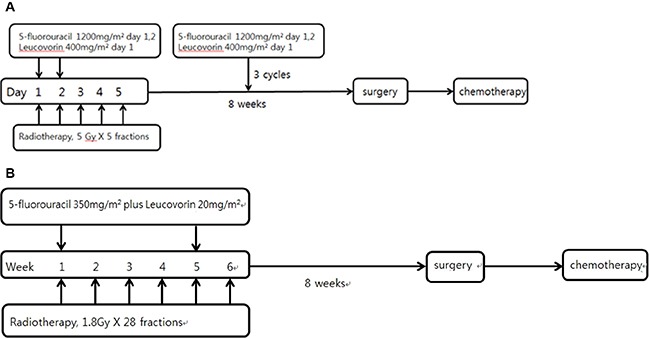
Treatment scheme of short-course CRT(A) and long-course CRT(B)

Surgery was performed 8 weeks after completion of CRT in both groups. Adjuvant chemotherapy was routinely recommended 4 weeks after surgery. Our Institutional Review Board approved this retrospective study. The treatment regimen is shown in Figure 1.

### Statistical analysis

All statistical analyses were performed using SPSS version 12.0 (Chicago, IL, USA). Between-group variables were compared using a chi-square test. Overall survival (OS) was defined as the time interval from the day of surgery to the day of death, if death occurred. The survival rate was estimated by the Kaplan-Meier method and compared between the groups with a log-rank test. A *p* value of < 0.05 in two-tailed tests was considered statistically significant.

## RESULTS

### Patient and tumor characteristics

Between October 2010 and April 2015, 72 patients with stage II or III rectal cancer underwent preoperative CRT at Gandong Kyung Hee University Hospital. The median follow-up period was 25 months and ranged from 3.0 to 58.0 months. Patient characteristics are summarized in Table 1. There were 14 (73.7%) patients under 70 years of age and 5 (26.3%) patients over 70 in the short-course CRT group, whereas there were 41 (77.4%) patients under 70 years of age and 12 (22.6%) patients over 70 in the long-course CRT group. There were 15 (78.9%) patients in stage cT3 and 4 (21.1%) patients in stage cT4 in the short-course CRT group, and 48 (90.6%) patients in stage cT3 and 5 (9.4%) patients in stage cT4 in the long-course CRT group. There were 6 (31.6%) cN0 patients and 13 (68.4%) cN+ patients in the short-course CRT group, whereas there were 12 (22.6%) cN0 patients and 41 cN+ (77.4%) patients in the long-course CRT group. There were no significant differences in any of these characteristics between the two groups.

**Table 1:** Patient characteristics (*n* = 72)

characteristic		Short course (*n* = 19)	Long course (*n*= 53)	*p*
Age (median 72 yr)	< 70	14 (73.7)	41 (77.4)	0.759
	≥ 70	5 (26.3)	12 (22.6)	
Gender	Male	10 (52.6)	38 (71.7)	0.161
	female	9 (47.4)	15 (28.3)	
cT classification	cT3	15 (78.9)	48 (90.6)	0.231
	cT4	4 (21.1)	5 (9.4)	
cN classification	cN0	6 (31.6)	12 (22.6)	0.539
	cN+	13 (68.4)	41 (77.4)	
Pretreatment CEA	≤ 5 ng/mL	12 (63.2)	31 (58.5)	0.790
	> 5 ng/mL	7 (36.8)	22 (41.5)	
Differentiation	Well	3 (15.8)	6 (11.4)	0.197
	Moderate	15 (78.9)	42 (79.2)	
	Poor	1 (5.3)	5 (9.4)	
Adjuvant chemotherapy	FL	11 (57.9)	33 (62.3)	0.222
	Capecitabine	0 (0.0)	6 (11.3)	
	Observation	8 (42.1)	14 (26.4)	

Data are presented as *n* (%).

Abbreviations: CEA = carcinoembryonic antigen; FL = 5-Fluouracil + Leucovorin.

### Tumor response after chemoradiotherapy

Correlations between clinopathologic factors and radiotherapy group are shown in Table 2. A ypCR was observed in 4 (21.1%) cases in the short-course CRT group and 7 (13.2%) cases in the long-course CRT group (*p* = 0.465). Downstaging was observed in 9 (47.4%) cases in the short-course CRT group and 14 (26.4%) cases in the long-course CRT group (*p* = 0.150).

**Table 2:** Correlations between clinicopathologic factors and the radiation groups

factor		Short course (*n* = 19)	Long course (*n* = 53)	*p*
Complete remission	Yes	4 (21.1)	7 (13.2)	0.465
	No	15 (78.9)	46 (86.8)	
Downstaging	Yes	9 (47.4)	14 (26.4)	0.150
	No	10 (52.6)	39 (79.6)	
LAR		17 (89.5)	50 (94.3)	0.602
APR		2 (10.5)	9 (5.7)	
CRM	Negative	17 (89.5)	47 (88.7)	0.925
	Positive	2 (10.5)	6 (11.3)	
Distance from anal verge	< 5 cm	12 (63.2)	18 (34.0)	0.033
	≥ 5 cm	7 (36.8)	65 (66.0)	
Local recurrence	Yes	1 (5.3)	1 (1.9)	0.442
	No	18 (94.7)	52 (98.1)	
Distant metastasis	Yes	1 (5.3)	12 (22.6)	0.162
	No	18 (94.7)	41 (77.4)	

Data are presented as *n* (%).

Abbreviations: LAR = low anterior resection; APR = abdominoperineal resection; CRM = circumferential resection margin.

Low anterior resection was performed on 17 (89.5%) patients in the short-course CRT group and 50 (94.3%) patients in the long-course CRT group (*p* = 0.602). Circumferential resection margin negative was observed in 17 (89.5%) cases in the short course CRT group and 47 (88.7%) cases in the long-course CRT group (*p* = 0.925).

### Recurrence and survival

Postoperative recurrence occurred in 15 patients, including both locoregional and distant recurrence. Locoregional recurrence (LRR) was seen in 1 (5.3%) patient at 28 months follow-up in the short-course CRT group and 1 (1.9%) patient at 9 months follow-up in the long-course CRT group (*p* = 0.442). Distant metastasis (DM) was seen in 1 (5.3%) patient at 5 months follow-up in the short-course CRT group and 12 (22.6%) patients at 32 months follow-up in the long-course CRT group (*p* = 0.162). The most common sites of DM were the lung and liver.

**Figure 2:**
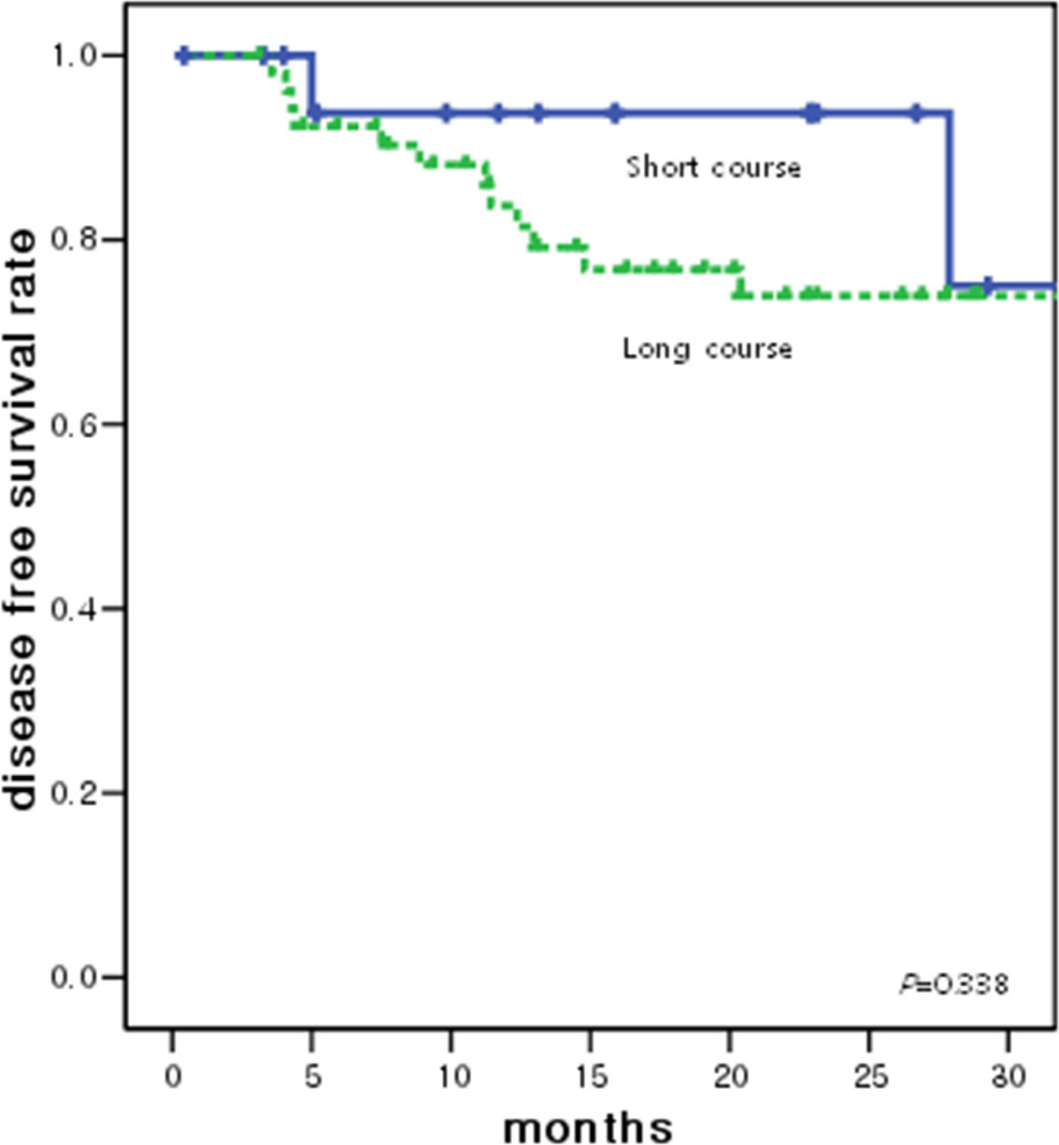
Disease free survival curve according to the radiotherapy groups The 2-year DFS rates of patients in the short-course CRT and long-course CRT groups were 93.8% and 74.0% (*p* = 0.338), respectively.

**Figure 3:**
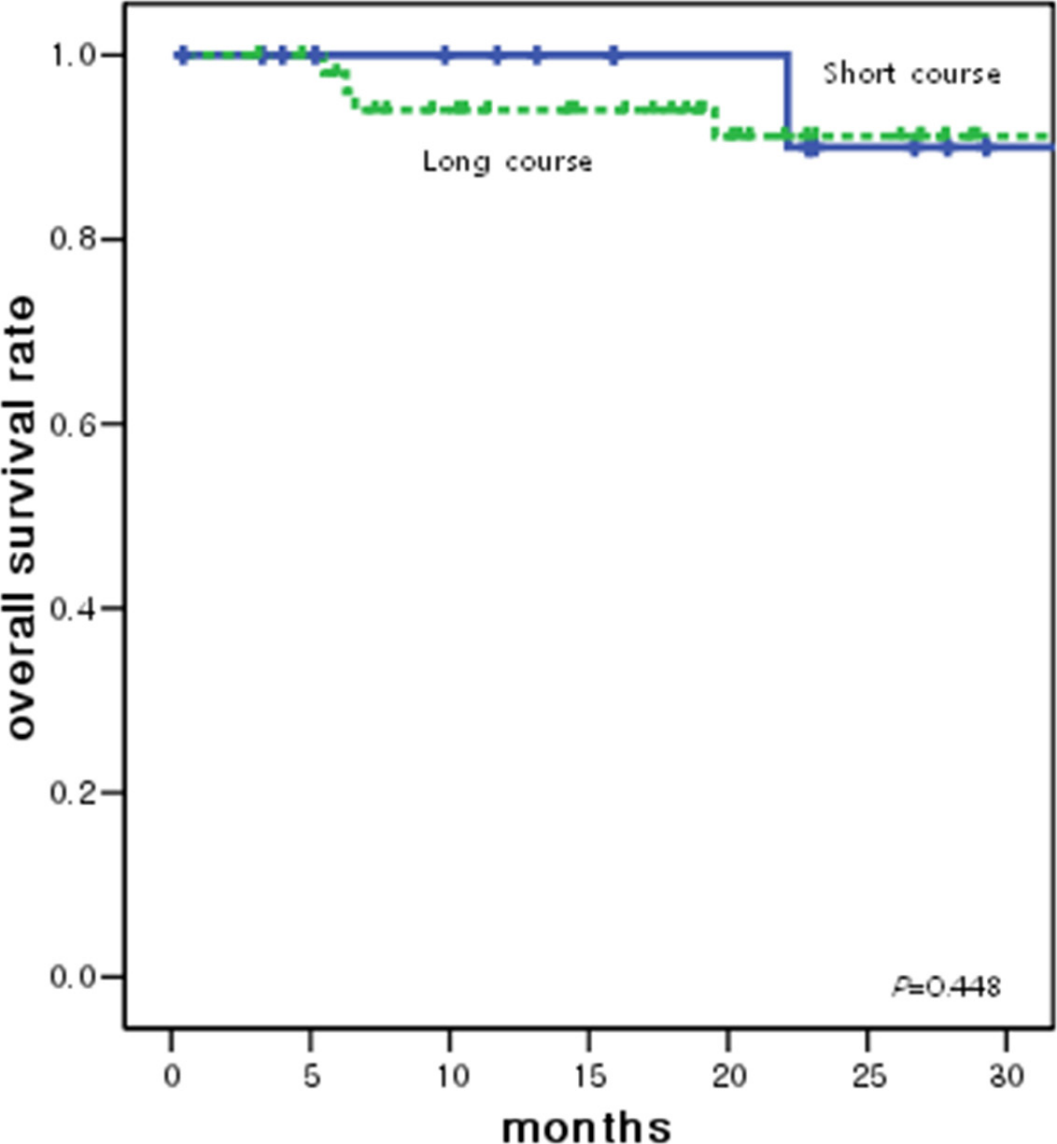
Overall survival curve according to the radiotherapy groups 2-year OS rates of patients in the short-course CRT and long-course CRT groups were 90.0% and 91.2% (*p* = 0.448), respectively.

The disease-free survival (DFS) and overall survival (OS) curves associated with both treatments are shown in Figures 2 and 3, respectively. The 2-year DFS rates of patients in the short-course CRT and long-course CRT groups were 93.8% and 74.0% (*p* = 0.338), respectively; 2-year OS rates were 90.0% and 91.2% (*p* = 0.448), respectively. Death was seen in 1 patient at 22 months follow-up and another patient at 39 months follow-up in the short-course CRT group and 4 patients at 24 months follow-up and 1 patient at 52 months follow-up in the long-course CRT group.

### Toxicity

Toxicity was scored according to the National Cancer Institute Common Terminology Criteria for Adverse Events, version 3.0. Severe (grade ≥ 3) toxicities are summarized in Table 3. Grade 4 anastomosis site leakage requiring surgical intervention was noted in 1 (5.6%) patient and grade 3 hematologic toxicity and bowel adhesion was noted in 1 (5.6%) patient in the short-course CRT group.

**Table 3:** Toxicities according to the radiation groups

	Short course		Long course	
	Grade 3	Grade 4	Grade 3	Grade 4
Leakage	3 (16.7)	1 (5.6)	5 (9.4)	1 (1.9)
Hematology	1 (5.6)	0 (0.0)	0 (0.0)	0 (0.0)
Diarrhea	0 (0.0)	0 (0.0)	1 (1.9)	0 (0.0)
Adhesion	1 (5.6)	0 (0.0)	1 (1.9)	0 (0.0)

Data are presented as *n* (%).

## DISCUSSION

The present study was conducted in order to evaluate the efficacy of concurrent chemotherapy with short-course radiotherapy followed by delayed surgery. Our results demonstrate that short-course CRT with delayed surgery led to pathologic responses and downstaging rates comparable to those of long-course CRT for patients with stage II or III rectal cancer. ypCR and downstaging rates were 4% and 47.4%, respectively, after short-course CRT, which did not significantly differ from the outcomes of long-course CRT.

Short-course radiotherapy followed by immediate surgery tends to have a high postoperative complication rate and lack of downstaging [11, 12]. Favorable effects of delayed surgery after short-course radiotherapy were reported in a recent investigation that examined whether the interval between preoperative short-course CRT and surgery for rectal cancer influenced outcomes [13]. An ongoing study is addressing the difference in downstaging achieved after long-course CRT and short-course radiotherapy followed by delayed surgery [9]. That trial randomly assigned 83 patients with rectal cancer to either short-course radiotherapy and delayed surgery or long-course CRT. The available results show that ypCR occurred in 1 (2.7%) patient in the short-course radiotherapy group and 6 (13.1%) patients in the long-course CRT group. Downstaging was observed in 8 (21.6%) patients in the short-course radiotherapy group vs. 18 (39.1%) patients in the long-course CRT group. Although it should be noted that our study had a small sample size, our results showed a higher ypCR and more downstaging in comparison with these other studies.

The above-mentioned review article asked whether the addition of chemotherapy to preoperative radiotherapy improved pathologic response and survival [14]. Four randomized trials were conducted that compare preoperative CRT with radiotherapy alone in locally advanced rectal cancer [10, 15–17]. The addition of chemotherapy to preoperative radiotherapy was shown to significantly increase local control and ypCR. However, the benefit of adding chemotherapy to short-course radiotherapy is not clear. The Korean Radiotherapy Oncology Group (KROG) 10–01 Phase II trial was designed to evaluate the efficacy of preoperative short-course radiotherapy plus concurrent chemotherapy followed by delayed surgery in patients with mid-to-distal locally advanced rectal cancer [18]. Preoperative FL and radiotherapy were administered on the same 5 days in that trial. ypCR was observed in only one patient. Downstaging occurred in 20 (28.2%) patients. Preoperative short-course CRT followed by delayed surgery was associated with poor pathologic responses compared with conventional long-course CRT. Compared with these results, the present study showed superior outcomes, with a downstaging rate of 47.4% and a ypCR rate of 21.1% in the short-course CRT group. We posit that concurrent chemotherapy with short-course radiotherapy and an additional 3 cycles of chemotherapy before surgery may be able to consolidate treatment without affecting outcomes. Although several short-course radiotherapy studies focused on concurrent chemotherapy for locally advanced rectal cancer, none of them addressed the effects of consolidated chemotherapy before surgery. Furthermore, chemotherapy is associated with toxicity. Only 1 patient (5.6%) in the short-course CRT group experienced grade 3 hematologic toxicity in the present study. So, we believe that chemotherapy with short-course radiotherapy and an additional 3 cycles of chemotherapy before surgery may be a feasible and relatively gentle treatment option.

DM was seen in 1 (5.3%) patient in the short-course CRT group and 12 (22.6%) patients in the long-course CRT group (*p* = 0.162). Although this difference is not statistically significant, the short-course CRT group showed a trend towards a lower risk of DM. We assume that a hypofractionated radiotherapy regimen has a role in the activation of the immune system. Evidence that has emerged over the last decade suggests an important role of radiotherapy in amplifying the antitumor immune response [19]. Recent clinical observations support the use of radiotherapy in preventing metastasis of tumors. A hypofractionated radiotherapy regimen may be associated with alterations in the tumor microenvironment that facilitate infiltration of host immune cells such as macrophages, dendritic cells, or tumor-antigen-specific cytotoxic T lymphocytes [20]. The hypothesis that hypofractionated radiotherapy regimens contribute to a decrease in DM warrants further investigation.

The current study demonstrates that preoperative short-course CRT and delayed surgery in patients with stage II or III rectal cancer has pathologic outcomes comparable to those seen after long-course CRT. However, the study has some limitations. The sample size was small (72 patients), and the study was conducted retrospectively. The adjuvant treatment groups were divided into an observation group and a chemotherapy group. Also, the chemotherapy regimen was chosen FL or capecitabine based on the discretion of the prescribing physician. There were 44 cases (61.1%) of LF and 6 cases (8.3%) of capecitabine for adjuvant chemotherapy. Despite these limitations, we identified meaningful benefits of a short-course CRT regimen about downstaging with ypCR.

In conclusion, preoperative short-course CRT and delayed surgery for patients with stage II or III rectal cancer demonstrated pathologic responses comparable to those seen with preoperative long-course CRT. Prospective studies are needed to investigate the difference between short- and long-course CRT in locally advanced rectal cancer patients.
